# Oral health profile and periodontal diseases awareness and knowledge among the jordanian population: a cross-sectional study

**DOI:** 10.1186/s12903-023-03203-8

**Published:** 2023-07-19

**Authors:** Mustafa Yousef Naser, Moath Momani, Abdallah Y Naser, Musab Ali Alarabeyat, Ahmad Mustafa Barakat Altarawneh, Ahmad Shaher Aladwan

**Affiliations:** 1 Prosthodontics Department, Royal Medical Services, Amman, Jordan; 2 Department of Applied Pharmaceutical Sciences and Clinical Pharmacy, Faculty of Pharmacy, Isra University, Amman, Jordan; 3 Department of Periodontics, Royal Medical Services, Amman, Jordan; 4 Department of Maxillofacial surgery, Royal Medical Services, Amman, Jordan

**Keywords:** Health, Jordan, Knowledge, Oral, Periodontics

## Abstract

**Objective:**

To explore the oral health profile and periodontal diseases awareness and knowledge among the Jordanian population. In addition, we aimed to identify predictors of good knowledge of periodontal diseases.

**Method:**

This was an online cross-sectional survey study that was conducted in Jordan between January and May 2022. A total of 13 item from the world health organisation (WHO) oral health questionnaire for adults were used to examine the oral health profile of our study participants. In addition, a previously developed questionnaire by Abdulbaqi et al. were adapted and used to examine participants’ knowledge about periodontal diseases. Binary logistic regression analysis was used to identify predictors of better knowledge of periodontal diseases.

**Results:**

This study involved 1,099 participants in total. More than half of them (61.1%) claimed that throughout the previous 12 months, they had experienced pain or discomfort in their mouths or teeth. Nearly half of the participants said their teeth and gums were in good or very good condition. 70.7% said they brush their teeth once or more per day. The vast majority of them (93.0%) claimed to brush their teeth using toothpaste that contained 61.9% fluoride. The most frequent cited cause for dental visits was pain or difficulty with teeth, gums, or mouth (36.3%), according to almost one-third of study participants who said they had visited a dentist during the previous six months. The most commonly reported problems that occurs frequently due to the state of the participants’ teeth or mouth were avoiding smiling because of teeth, feeling embarrassed due to appearance of teeth, and having difficulty in biting foods with 11.0%, 10.2%, and 9.0%, respectively. Tea with sugar (16.5%) was the most frequently reported beverage as being consumed frequently on a daily basis. The most popular tobacco product to be smoked often on a daily basis was cigarettes (21.6%). For periodontitis knowledge questions, the percentage of accurate responses ranged from 32.3 to 55.8%. The majority of participants (55.8%) were able to recognize that poor oral hygiene is one of the most frequent causes of malodor, whereas the least number of participants (32.3%) were able to recognize that improper teeth brushing is a frequent cause of gingival recession.

**Conclusion:**

The average degree of periodontitis knowledge among Jordanians was moderate. Along with it, there were modest oral hygiene practices. In order to prevent further oral complications that have a detrimental influence on patients’ quality of life, educational campaigns are required to increase public awareness of knowledge and practices in terms of proper oral hygiene and periodontitis.

## Introduction

Maintaining good oral hygiene has been a universal human necessity that has afflicted humans since the dawn of history. It can reflect the overall well-being of an individual, regardless of their social status [1]. Recently, the significance of oral hygiene and care has focused on associations between periodontal health and general health, as many studies have investigated the potential links between poor oral care and various chronic diseases, such as cardiovascular disease, respiratory diseases, and diabetes [1–4].

Periodontal disease is a chronic microbial-induced inflammatory process that affects one or more components of the periodontium. It prompts the destruction of tooth-supporting tissues such as gingiva, periodontal ligament, cementum, and alveolar bone, and it continues to be a significant factor in tooth loss among adults in both developed and developing nations [5, 6]. There are different forms of periodontal problems that can arise, such as untreated caries, which frequently causes acute infection alongside dental pain and discomfort, which is common among children and teenagers. In many developed countries, the extraction of carious teeth under general anesthesia is the main reason for hospitalization of young children [7]. Even for older people, oral diseases result in specific challenges related to pain, impaired chewing and eating ability, or even nutritional deficiencies [8].

In Western industrialized countries, most of the population considers oral hygiene beneficial to oral health. Nevertheless, oral health practices continue to be strongly related to social factors, living circumstances, and general lifestyle [9]. Furthermore, oral habits and oral hygiene practices adopted by individuals can have either a negative or positive impact on oral health, which continues to persist as a highly widespread disease that can affect up to 90% of the worldwide population, as it collectively affected 3.9 billion people in 2010 [6, 10, 11]. Despite the fact that most oral conditions are not life-threatening, quality of life can be affected by poor oral health [12].

Oral health knowledge related to periodontal diseases has a major role in the treatment and prevention of the disease among children, adolescents, and adults, including university students [13, 14]. There are limited studies in Jordan that examined the oral health status and periodontal diseases awareness and knowledge. Consequently, the purpose of this study was to investigate the knowledge and awareness of periodontal disease and oral health among Jordanians. In addition, we aimed to examine the oral health profile among the Jordanian population. This study provides data for future research and allows for comparisons of oral health knowledge in other nations.

## Method

### Study design and settings

From January to May 2022, an online cross-sectional survey was undertaken in Jordan to examine oral health profile and periodontal diseases awareness and knowledge among the Jordanian population.

### Sampling procedure

This investigation’s sample was selected using a technique known as convenience sampling technique. This study included voluntary participants who met the inclusion criteria and were deemed eligible. On the first page of the questionnaire, patients were presented with an informed consent form and given the option to continue or withdraw from the study. To ensure that the patients comprehended the significance of their participation, the study’s objectives were presented in detail. The inclusion criteria were specified in the study’s invitation letter.

### Study population and recruitment

The inclusion criteria for this study’s sample population included Jordanian adults aged 18 and older. There were no age or gender-based criteria for exclusion. Any participant who did not meet the study inclusion criteria was excluded from this study. The URL to the survey was disseminated on social media platforms (Facebook, Instagram, and SnapChate) to invite interested individuals to participate.

### Study tool

This questionnaire was used to examine oral health profile and periodontal diseases awareness and knowledge among the Jordanian population. A total of 13 items from the World Health Organization (WHO) oral health questionnaire for adults were used to examine the oral health profile of our study participants [15]. The Adult Oral Health Questionnaire is a tool used to assess the oral health status and related behaviors of adults. It is a questionnaire designed to collect information regarding an individual’s oral hygiene practices, dental behaviors, and oral health in general. Typically, the questionnaire encompasses a variety of oral health-related topics, such as dental visits, frequency of brushing, tobacco use, alcohol consumption, diet, and oral hygiene practices. In addition, a previously developed questionnaire by Abdulbaqi et al. were adapted and used to examine participants’ knowledge about periodontal diseases (7-items) [16]. Knowledge items examined participants’ awareness of periodontal diseases risk factors, clinical characteristics, and management. For each correct answer, the participants were given a score of one. The maximum expected score is seven. The higher the score the more knowledgeable the individual of periodontal diseases. In addition, the questionnaire took information regarding the respondent’s demographic information such as age, gender, level of education, employment, monthly income level, and history of chronic diseases.

### Sample size

The minimum required sample size was 385 individuals using a 95% confidence interval, a 0.5 standard deviation (SD), and a 5% margin of error.

### Statistical analysis

This study’s data were analyzed using version 27 of the Statistical Package for Social Science program. Categorical variables were measured using frequency and percentage. Continuous variables were measured using mean and standard deviation (SD) as the data were normally distributed. Binary logistic regression analysis was used to identify predictors of better knowledge of periodontal diseases. The dummy variable for the logistic regression was identified using the mean knowledge score of the study participants as a cut-off point (which was 3.0 (SD: 1.8)). A confidence interval (CI) of 95% (p < 0.05) was applied to represent the statistical significance of the results, and the level of significance was assigned as 5%.

## Results

### Participants’ demographic characteristics

A total of 1,099 participants were involved in this study. More than half of them (55.1%) were females. More than half of them (59.0%) were aged 26 years and below and were single (62.1%). The majority of them (73.3%) reported that they hold bachelor degree. Almost 40.0% of the participants were university students. More than half of them (63.2%) reported that their monthly income is 500 Jordanian dinar (JD) and lower. Table 1 below presents the demographic characteristics of the study participants.

**Table 1:** Participants’ demographic characteristics

Variable	Frequency	Percentage (%)
**Gender**		
Females	605	55.1
**Age group**		
18–20 years	188	17.1
21–23 years	307	27.9
24–26 years	154	14.0
27–30 years	137	12.5
30–35 years	126	11.5
36–40 years	63	5.7
41 years and over	124	11.3
**Marital status**		
Single	683	62.1
Married	364	33.1
Divorced	36	3.3
Widowed	16	1.5
**Education level**		
High school level or lower	203	18.5
Bachelor degree	806	73.3
Higher education	90	8.2
**Employment status**		
Retired	48	4.4
Unemployed	223	20.3
Student	440	40.0
Working in the healthcare sector	173	15.7
Working outside the healthcare sector	215	19.6
**Monthly income level**		
Less than 500 JD	689	63.2
500–1000 JD	301	27.6
1000–1500 JD	69	6.3
1500 JD and above	31	2.8
**History of chronic diseases**		
Yes	140	12.7

JD: Jordanian Dinar

### Oral health profile of the study participants

Table 2 below presents oral health profile of the study participants. The overwhelming majority of participants (79.1%) reported having 20 or more natural teeth. More than half of them (61.1%) reported that their teeth or mouth had caused them pain or distress in the previous year. The vast majority (82.5%) of participants who reported having no natural teeth reported wearing partial dentures. Nearly half of the participants rated the condition of their teeth and gums as satisfactory or excellent. The majority of respondents (70.7%) indicated that they brush their teeth at least once per day. The toothbrush was the most commonly reported teeth-cleaning device (90.7%). The vast majority of them (93%) reported using toothpaste to clean their teeth, and 61.9% of those toothpastes contained fluoride. Nearly one-third of the study participants reported that they had visited their dentist within the past six months, with tooth, periodontal, or mouth discomfort being the most common reason (36.3%). The most frequently reported problems that frequently occur due to the state of the participants’ teeth or mouth were avoiding beaming because of teeth, feeling humiliated by the appearance of teeth, and having difficulty biting food, with 11.0%, 10.2%, and 9.0%, respectively. Tea with sugar was the most frequently reported beverage consumed frequently on a daily basis (16.5%). The majority of respondents (21.6%) who frequently smoked tobacco products on a daily basis reported smoking cigarettes.

**Table 2:** Oral health profile of the study participants

Variable							Frequency		Percentage (%)
**How many natural teeth do you have?**									
No natural teeth							40		3.9
1–9 teeth							45		4.4
10–19 teeth							129		12.6
20 teeth or more							809		79.1
**During the past 12 months, did your teeth or mouth cause any pain or discomfort?**									
Yes							623		61.1
**Do you have any removable dentures? (n = 40)**									
A partial denture (Yes)							34		85.0
A full upper denture (Yes)							3		7.5
A full lower denture (Yes)							3		7.5
**How would you describe the state of your ….**		**Very poor**		**Poor**		**Average**	**Good**	**Very good**	
Teeth		3.8		8.7		28.1	37.4	22.0	
Gums		3.3		9.7		25.6	37.4	23.9	
**How often do you clean your teeth?** (n = 1,023)									
Never							64		6.3
Once a month							36		3.5
2–3 times a month							40		3.9
Once a week							63		6.2
2–6 times a week							97		9.5
Once a day							374		36.6
Twice or more a day							349		34.1
**Do you use any of the following to clean your teeth?** (n = 1,023)									
Toothbrush							928		90.7
Wooden toothpicks							364		35.6
Plastic toothpicks							207		20.2
Thread (dental floss)							265		25.9
Charcoal							124		12.1
Chewstick/miswak							308		30.1
**Do you use toothpaste to clean your teeth?** (Yes) (n = 1,023)							951		93.0
**Do you use a toothpaste that contains fluoride?** (Yes) (n = 1,023)							633		61.9
**How long is it since you last saw a dentist?** (n = 1,023)									
Less than 6 months							376		36.8
6–12 months							204		19.9
1–2 years							204		19.9
2–5 years							92		9.0
More than 5 years							147		14.4
**What was the reason of your last visit to the dentist?** (n = 1,023)									
Consultation/advise							102		10.0
Pain or trouble with teeth, gums or mouth							371		36.3
Treatment/ follow-up treatment							219		21.4
Routine check-up/treatment							121		11.8
Don’t know/don’t remember							210		20.5
**Because of the state of your teeth or mouth, how often have you experienced any of the following problems during the past 12 months?**			**Don’t know**		**No**	**Sometimes**	**Fairly often**	**Very often**	
Difficulty in biting foods			10.7		56.0	24.3	6.3	2.7	
Difficulty chewing foods			7.3		61.1	22.7	6.4	2.5	
Difficulty with speech/trouble pronouncing words			9.4		72.8	11.9	4.0	1.9	
Dry mouth			9.3		58.3	24.3	5.4	2.7	
Felt embarrassed due to appearance of teeth			7.2		62.9	19.7	5.9	4.3	
Felt tense because of problems with teeth or mouth			7.6		61.6	21.9	4.9	4.0	
Have avoided smiling because of teeth			5.1		66.1	17.8	6.8	4.2	
Had sleep that is often interrupted			7.6		63.6	20.0	6.0	2.7	
Have taken days off work			6.9		72.8	15.1	3.4	1.8	
Difficulty doing usual activities			7.7		70.4	15.2	3.3	3.4	
Felt less tolerant of spouse or people who are close to you			15.6		70.3	9.9	2.1	2.2	
Have reduced participation in social activities			12.4		72.2	10.3	2.5	2.5	
**How often do you eat or drink any of the following foods, even in small quantities?**	**Seldom/never**		**Several times a month**		**Once a week**	**Several times a week**	**Every day**	**Several times a day**	
Fresh fruit	6.3		4.0		12.3	29.1	33.4	14.9	
Biscuits, cakes, cream cakes	10.1		6.2		14.3	32.6	27.2	9.8	
Sweet pies, buns	12.8		7.9		19.0	33.9	19.6	6.8	
Jam or honey	31.4		12.5		18.5	20.9	12.4	4.3	
Chewing gum containing sugar	25.0		8.7		14.5	20.5	20.9	10.4	
Sweets/candy	13.8		12.5		18.8	28.9	19.6	6.5	
Lemonade, Coca Cola or other soft drinks	19.9		8.2		13.9	24.1	23.8	10.1	
Tea with sugar	20.6		5.5		10.3	17.8	29.3	16.5	
Coffee with sugar	48.1		4.5		7.2	11.1	18.2	10.9	
	**Never**		**Seldom**		**Several times a month**	**Once a week**	**Several times a week**	**Every day**	
Cigarettes	67.5		4.8		1.4	2.2	2.5	21.6	
Cigars	81.1		7.4		2.0	2.2	2.5	4.7	
A pipe	59.9		9.9		5.3	6.5	6.4	12.0	
E-cigarettes	72.8		6.9		2.8	3.2	3.9	10.3	

### Knowledge and awareness of periodontitis

Overall, the study participants demonstrated a weak level of knowledge of periodontal diseases, with a mean knowledge score of 3.0 (1.8) out of 7, which is equal to 42.9% of the maximum score. The proportion of correct answers for knowledge items regarding periodontitis ranged between 32.3% and 55.8%. The highest proportion of the participants were able to identify that poor oral hygiene is one of the most common reason for malodour (55.8%) and the lowest proportion of the participants were able to identify that faulty tooth brushing is the common reason for gingival recession (32.3%). For further details on the participants’ answers for the knowledge items, refer to Table 3.

**Table 3:** Knowledge and awareness of periodontitis

Variable	Frequency	Percentage (%)
**What is the most common reason for gingival bleeding?** (n = 1,013)		
Vitamin C deficiency	170	16.8
Genetics	104	10.3
Poor oral hygiene*	546	53.9
Don’t know	193	19.1
**What is the most common reason for malodour?** (n = 1,013)		
Alcohol drinking	116	11.5
Smoking	239	23.6
Poor oral hygiene*	565	55.8
Type of food	93	9.2
**What is the most common reason for increasing tooth mobility?** (n = 1,013)		
Diabetes mellitus	148	14.6
Injury/trauma	125	12.3
Periodontal disease*	478	47.2
Aging	262	25.9
**Do you think that teeth hypersensitivity can be treated?** (n = 1,013)		
No	55	5.4
Yes*	535	52.8
Maybe	294	29.0
Don’t know	129	12.7
**Do you think that periodontal health could affect general health?** (n = 1,013)		
No	94	9.3
Yes*	498	49.2
Maybe	284	28.0
Don’t know	137	13.5
**What is the most common reason for gingival recession?** (n = 1,013)		
Injury	197	19.4
Diabetes mellitus	103	10.2
Faulty tooth brushing*	327	32.3
Habits	386	38.1
**Do you think that the prevalence of periodontal disease increases after 35 years’ age?** (n = 1,013)		
No	95	9.4
Yes*	383	37.8
Maybe	349	34.5
Don’t know	186	18.4

* right answer

Binary logistic regression analysis identified that females, young participants aged 21–23 years, those who hold bachelor’s degree, and those who work in the healthcare sector were more likely to be knowledgeable of periodontal diseases compared to others (p < 0.05). Table 4 presents predictors of better knowledge of periodontal diseases identified using binary logistic regression analysis.

**Table 4:** Predictors of better knowledge of periodontal diseases

Variable	Odds ratio of having better knowledge of periodontal diseases (95% confidence interval)	P-value
**Gender**		
Males (Reference group)	1.00	
Females	1.32 (1.03–1.68)	0.028*
**Age group**		
18–20 years (Reference group)	1.00	
21–23 years	1.45 (1.10–1.92)	0.009**
24–26 years	1.09 (0.77–1.55)	0.627
27–30 years	1.25 (0.86–1.83)	0.242
30–35 years	1.22 (0.82–1.80)	0.327
36–40 years	0.76 (0.45–1.27)	0.289
41 years and over	0.78 (0.53–1.13)	0.188
**Marital status**		
Single (Reference group)	1.00	
Married	0.95 (0.73–1.22)	0.671
Divorced	0.86 (0.44–1.68)	0.657
Widowed	0.47 (0.18–1.28)	0.141
**Education level**		
High school level or lower (Reference group)	1.00	
Bachelor degree	1.94 (1.48–2.54)	< 0.001***
Higher education	0.87 (0.56–1.35)	0.543
**Employment status**		
Retired (Reference group)	1.00	
Unemployed	0.57 (0.43–0.77)	< 0.001***
Student	1.35 (0.95–1.90)	0.090
Working in the healthcare sector	1.33 (1.04–1.71)	0.025*
Working outside the healthcare sector	1.19 (0.87–1.62)	0.276
**Monthly income level**		
Less than 500 JD (Reference group)	1.00	
500–1000 JD	1.02 (0.77–1.33)	0.916
1000–1500 JD	1.02 (0.62–1.69)	0.937
1500 JD and above	1.30 (0.61–2.80)	0.496
**History of chronic diseases**		
No (Reference group)	1.00	
Yes	0.75 (0.53–1.08)	0.120

*p < 0.05;**p < 0.01; ***p < 0.001

## Discussion

The key findings of this study are: (1) more than half of the study participants stated that their teeth or mouth caused pain or discomfort during the past 12 months, (2) the majority of the participants reported that they clean their teeth once or more per day, (3) the vast majority of them reported that they use toothpaste to clean their teeth of which more than half contained fluoride. Almost one-third of the study participants reported that they visited their dentist within the past 6 months, and (4) the participants’ knowledge of periodontitis was low to moderate.

This study was an online-based survey that assessed the level of awareness and knowledge on oral health profiles and periodontal health among the Jordanian population using a well-established online questionnaire to examine the oral health profiles of our study participants. Studies have proven the advantages of online surveys, such as the ability of the internet to access a wider group base and save time and effort for researchers and participants [14]. In this study, a total representative sample of 1,099 participants was found adequate to give some insights into the level of knowledge and awareness of periodontal health among the targeted population and provide baseline data for the study’s objectives. Similar studies in different nations have also been conducted in the past to estimate the knowledge, awareness, and behavior of different groups, including professionals, students, and populations.

Overall, the study participants demonstrated a weak level of knowledge of periodontal diseases. A previous study in Jordan conducted by Alzammam and Almalki examined the knowledge and awareness of periodontal diseases among university students and found that uiversity students lacked sufficient knowledge of the causes of periodontal illnesses and the function of conventional therapy in preserving excellent oral health by halting the inflammatory process [17]. Another study in Iraq evaluated oral health and periodontal diseases awareness among the general public and reported low to moderate levels of awareness about periodontal diseases [16]. Furthermore, another study conducted in Saudi Arabia examined the periodontal health knowledge and awareness of a group of dental patients and reported a low level of awareness regarding the precise cause of periodontal disease [18].

Our assessment of the study participants’ knowledge of periodontal health indicated that 61.1% of participants stated that their teeth or mouth caused pain or discomfort during the past 12 months, which was higher than what was reported in previous literature. Compared to another study conducted in Tanzania, around 59% of participants reported suffering from oral pain and/or discomfort during the last 12 months that preceded the study [19]. In another study conducted in the United States, the city of Toronto showed that 53% of participants had experienced some pain or discomfort in the 4 weeks prior to the completion of the questionnaire [20]. Furthermore, another study conducted in the US on Special Olympic athletes reported that around 14% reported having oral pain on screening day; however, it also indicated that athletes with untreated caries (30%) were 1.70 times more likely to also report oral pain at the time of their screening [21]. In Nepal, a study was conducted on school children, and around 31% of participants reported suffering from oral pain [22]. Moreover, according to a study conducted in Sri Lanka on children as well, 25% of the children had experienced oral pain in the past 2 months, and almost 45% stated that the pain was severe. While 31% of the parents reported that their child had experienced oral pain within the same period [23]. In France, a study on oral health showed that the prevalence of sensitive teeth, as its symptoms can be characterized by oral pain and discomfort, during the previous 12 months reported in the sample was 42.2% [24, 25]. Jordanians have experienced oral pain slightly more than what was reported in previous studies from other countries. Moreover, it showed that children experienced less oral pain and discomfort during the study periods. Oral pain and discomfort can be a result of different factors, including psychological, behavioral, systemic, and inflammatory [26, 27], and they also vary in severity [20]. In addition, dental pain is the most common in the orofacial area [28], and as our participants reported, it can be the cause of many difficulties in biting and chewing and even cause stress and interrupt sleep.

One more aspect we aimed to assess was the prevalence of tooth-brushing daily, and we found that the majority of the Jordanians (70.7%) reported that they clean their teeth once or more per day, which shows a great percentage and can reflect the awareness of oral hygiene among Jordanians. According to a study conducted in Pakistan, in a medical school, the majority of students brush their teeth daily, with an overall percentage of 92.6% [29]. In India and among schoolchildren, the results show that 30.5% of the total sample cleaned their teeth twice or more daily [30]. Moreover, in Europe’s specially developed nations, a study based in 26 countries showed that the highest prevalence of tooth-brushing more than once a day was in Switzerland, with a percent of 80% for boys and 89% for girls, followed by Germany and the Netherlands. However, the overall lowest prevalence was in Malta, with a percent of 16% for boys and 26% for girls, and in all countries, the prevalence of tooth-brushing was higher in girls than boys [31]. Also, compared to a study conducted in France, tooth brushing frequency at least once per day was high, at around 93%, which was 1297 out of 1395 participants, showing a very high number compared to other nations [24]. Moreover, in Germany, a study showed that the majority of the participants (79.6%) brushed their teeth twice daily, and 11.7% and 8.7% brushed their teeth once and more than twice, respectively [32]. In conclusion, tooth-brushing has a direct relationship with oral health, which varies according to frequency, duration, technique, and toothpaste used [9]. However, faulty tooth-brushing can cause serious problems such as toothbrush abrasion [33], gum bleeding, and gingival recession [34–36] and in our study, 90.7% of participants used toothbrushes as their main tool for teeth cleaning. Furthermore, this indicates that developed nations have a higher prevalence of teeth cleaning than developing countries, and Jordanians showed great awareness and practice of teeth cleaning and oral hygiene.

The key to maintaining good oral hygiene and a healthy dentition and for sufficient and effective prevention or reduction of dental caries and gingivitis is dental plaque removal by tooth-brushing using a fluoride toothpaste, as it is effective against bacterial plaque, which can be the main reason for various periodontal issues such as dental canes, gingivitis, and periodontitis [9]. Regarding that, 61.9% of participants used toothpaste containing fluoride, which is what the vast majority of other populations used, as mentioned below. In Sweden, for example, the vast majority (98%) used fluoride toothpaste [37], and in a medical school in Pakistan, a percentage of 59.1% of the participants preferred to use toothpaste that contains fluoride [29].

Furthermore, one of the recent studies conducted in New Zealand showed that 79.9% of children and 84.9% of adults used standard fluoride toothpaste [38]. In Ireland, a cross-sectional study showed that more than half of participants used low-fluoride toothpaste [39]. Another cross-sectional study based in Japan showed that non-fluoride toothpaste users accounted for 5.1% of all toothpaste users [40]. This indicates that the majority of people all over the world use fluoride toothpaste, in both developing and developed countries. Nevertheless, fluoride is known for its numerous benefits in caries prevention, and it has been used over the past 50 years in different forms such as toothpaste, fluoride water, and mouthwash. However, some risk factors might arise from the high exposure to fluoride, especially dental fluorosis, which is a condition associated with enamel development [41, 42] and many dentists recommend parents use an age-related amount of toothpaste and keep an eye on fluoride concentrations [43, 44].

Our study also showed that almost one-third of the participants (36.8%) reported visiting their dentist within the past 6 months, which can indicate both good oral health and fewer check-ups; however, in another study conducted in 26 countries across Asia, Africa, and the United States, 25.6% and 16.3% reported visiting the doctor twice and once a year, respectively [45]. In another study conducted in Tanzania, 60.3% of participants reported that they had never attended a dental facility [46]. In Nigeria, 31.7% of participants visited a dental facility [47]. Moreover, in developed countries such as Italy [48] and France [24], the percentage of participants visiting the dentist was 33.3% in the past year and 37.3% in the past 6 months, respectively. In middle-income countries such as Turkey, almost one-third of participants visited the dentist for preventive treatment in the last year [49]. Another study conducted in India reported that 48% of participants had never visited the dentist before, which accounts for almost half of the participants [1]. In brief, the reasons for participants last visit to the dentist varied, with the highest percentage being 36.3% because of pain with teeth, gums, or mouth, followed by seeking treatment, routine check-ups, and consultations, which indicated a majority of “treatment seeking visitors” rather than “consultation and prevention seeking visitors”. Overall, Jordanians have a very similar pattern regarding the frequency of visiting the dentist and the reasons for doing so compared to other nations worldwide.

As the title of our study suggests, we aimed to estimate the knowledge of oral health among Jordanians, and the proportion of correct answers ranged between 32.3% and 55.8%. Which suggests a low to moderate indication of the participants’ knowledge. In comparison, in a study conducted in Al Karak, a city south-west of Amman, the capital, the mean knowledge among secondary school students was 60.8% [50]. Moreover, in a study conducted in Qatar on the awareness of oral health among children, only 25.8% reported a high level of knowledge [51]. Furthermore, in the United Arab Emirates, the percentage of correct answers among adults was 62% [52].

In another study conducted in Tanzania, the percentage regarding different aspects of periodontal health was between 26.5% and 68.6% [46]. In India, they found a huge problem due to a lack of periodontal awareness, as 62% of participants reported that oral health is not associated with general health [1]. In addition, a study in Malaysia indicated that among secondary school students, most participants scored below the mean, with only 43.5% scoring above it [53]. In another country in Africa, Nigeria, the overall findings showed that 84.3% of participants had a good view of oral health [54]. Jordanians correct answers regarding dental aspects were positive; however, in the relationship between age and periodontal disease, more than one-third weren’t sure about the relationship. In other aspects related to malodour, gingival bleeding, tooth mobility, and hypersensitivity, almost more than half of the participants’ answers were correct, indicating a positive overall view.

Halitosis, or malodour, is defined as the unpleasant breath smell that can interfere with a person’s confidence and image socially, and it has many reasons, including periodontal disease, certain types of food, and poor oral hygiene and practices [55, 56]. In a study done on social relations and breath odour, 75% of participants reported being told about their breath odour, which reflected on their self-confidence [57]. In our study, more than half of participants (55.8%) identified poor hygiene as the most common reason for malodour. In other studies, estimating the participants’ suggestions on the reasons, in Saudi Arabia, 78.1% of all female participants suggested that not brushing teeth was the most common reason, which indicated bad oral hygiene [55]. In another study also conducted in Saudi Arabia, the participants suggested that smelly food accounts for 84% of bad breath reasons, followed by gum diseases, and the least caffeinated drinks [58]. We also spotted the light on the participants’ suggestions of common reasons for gingival recession; the participants answers varied, with the highest percentage being faulty tooth-brushing (32.3%), which shows a good but still low overall awareness of the reason for the other answers. Gingival recession is characterized by the apical shift of the marginal gingiva from its normal position on the crown of the tooth to levels on the root surface beyond the cemento-enamel junction. When it comes to the causes, some studies on its etiology found that it can be caused by dental plaque accumulations and faulty tooth- brushing, among other systemic reasons [34–36].

In our study, females, young participants aged 21–23 years, those who hold bachelor’s degree, and those who work in the healthcare sector were more likely to be knowledgeable of periodontal diseases. A previous research in Iraq found that individuals aged 45 possessed greater levels of knowledge [16]. This may be explained by the accumulation of dental knowledge and experience over a lifetime, which may encourage elderly individuals to preserve their remaining teeth. In addition, the Iraqi study revealed that females demonstrated significantly higher oral health awareness than their male counterparts, particularly regarding daily tooth care practices such as frequency of brushing, use of mouthwashes, and knowledge of causes of teeth discoloration that may compromise their esthetics [16]. In accordance with studies conducted in Spain and the United States that utilized a comprehensive measure of oral hygiene knowledge and in which females exhibited higher oral health knowledge levels than males [59–61], these results indicate that females have a greater understanding of oral hygiene than males. In addition, confirming our study findings, according to the Iraqi study, employed individuals displayed a higher level of oral health awareness than unemployed individuals. Indeed, the stress of unemployment and a lack of consistent income can have a negative impact on life quality, which can manifest itself in poor oral hygiene, which can be exacerbated by the expense of visiting a dentist [16].

This study has limitations. The cross-sectional survey design of the research itself constrained our ability to establish a causal relationship between study variables. Self-administered online surveys are susceptible to social desirability bias and may not represent the situation accurately. Using a convenient sampling method may have limited the generalizability of our study’s results. Therefore, our findings should be carefully interpreted.

## Conclusion

Weak knowledge of periodontal diseases was demonstrated among the Jordanian population. At the same time, good oral health and practices was reported. Further studies are warranted using more generalizable sampling techniques. Dentists are advised to educate their patients further on periodontal diseases and their oral health and hygiene and emphasise on the important of this knowledge and practices on their health outcomes. It is recommended to educate and engage the community on the significance of oral health and periodontal disease prevention. This includes launching oral health education programs and collaborating with local healthcare providers in the development of community-wide awareness campaigns using a variety of communication channels, including posters, brochures, pamphlets, social media, and local publications.

## Data Availability

The datasets used and/or analysed during the current study are available from the corresponding author on reasonable request.

## Abbreviations

JD: Jordanian dinar
CI: confidence interval
